# The radical scavenging activity of 1-methyl-1,4-dihydronicotinamide: theoretical insights into the mechanism, kinetics and solvent effects†

**DOI:** 10.1039/d4ra07184k

**Published:** 2024-11-20

**Authors:** Quan V. Vo, Nguyen Thi Hoa, Adam Mechler

**Affiliations:** a The University of Danang – University of Technology and Education Danang 550000 Vietnam vvquan@ute.udn.vn; b Department of Biochemistry and Chemistry, La Trobe University Victoria 3086 Australia a.mechler@latrobe.edu.au

## Abstract

1,4-Dihydronicotinamide derivatives, including 1-methyl-1,4-dihydronicotinamide (MNAH), are derivatives of the active center of nicotinamide coenzyme (NADH) and are therefore potent radical scavengers. MNAH serves as a useful model of NADH that allows for modeling studies to address the activity of this important biomolecule. In this work, MNAH activity was evaluated against typical free radicals using quantum chemical calculations in physiological environments, with a secondary aim of comparing activity against two physiologically relevant radicals of markedly different stability, HO˙, and HOO˙, to establish which of these is a better model for assessing antioxidant capacity in physiological environments. The HO˙ + MNAH reaction exhibited diffusion-limited overall rate constants in all media, including the gas phase. The HOO˙ antiradical activity of MNAH was also good, with overall rate constants of 2.00 × 10^4^ and 2.44 × 10^6^ M^−1^ s^−1^, in lipid and aqueous media, respectively. The calculated rate constant in water (*k*_overall_(MNAH + HOO˙) = 3.84 × 10^5^ M^−1^ s^−1^, pH = 5.6) is in good agreement with the experimental data (*k*_exp_(NADH + HOO˙) = (1.8 ± 0.2)×10^5^ M^−1^ s^−1^). In terms of mechanism, the H-abstraction of the C4–H bond characterized the HOO˙ radical scavenging activity of MNAH, whereas HO˙ could react with MNAH at several sites and following either of SET (in polar media), RAF, and FHT reactions, which could be ascribed to the high reactivity of HO˙. For this reason the results suggest that activity against HOO˙ is a better basis for comparison of anti-radical potential. In the broader context, the HOO˙ scavanging activity of MNAH is better than that of reference antioxidants such as *trans*-resveratrol and ascorbic acid in the nonpolar environment, and Trolox in the aqueous physiological environment. Therefore, in the physiological environment, MNAH functions as a highly effective radical scavenger.

## Abstract 2

In physiological environments, 1-methyl-1,4-dihydronicotinamide (MNAH), an active center of the nicotinamide coenzyme (NADH), demonstrated significant radical scavenging activity.

## Graphical abstract

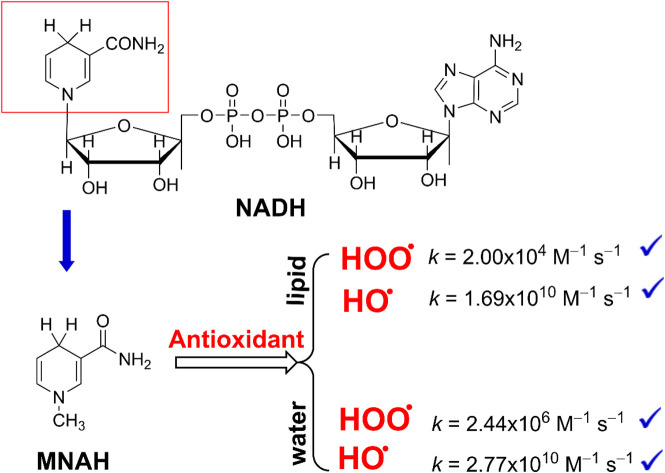

## 1. Introduction

Reactive oxygen species (ROS) are molecules that are extremely reactive and are primarily produced by the mitochondrial electron transport chain.^1^ Under normal circumstances, ROS are generated as natural biproducts of normal metabolic processes and also serve a variety of functions in healthy physiological processes. For example, they activate signaling pathways to initiate biological processes as “secondary messengers”.^2^ Oxidative stress is the result of an imbalance between the antioxidant defense system and ROS production.^3^ It is also crucial to preserve the equilibrium of ROS in bone homeostasis and pathology.^4^ Nicotinamide coenzyme (NADH) is a natural redox factor that is crucial for the reduction of ROS; it is a ubiquitous hydride and electron source that participates in a diverse array of biochemical processes that occur *in vivo*.^5–7^ The mechanism of oxidoreductase primarily relies on the cycling of nicotinamide adenine dinucleotide (NAD) and its reduced form, NADH. The NAD redox pair (NAD^+^/NADH) serves as a coenzyme essential for oxidoreductase metabolism.^8–12^

**Fig. 1:**
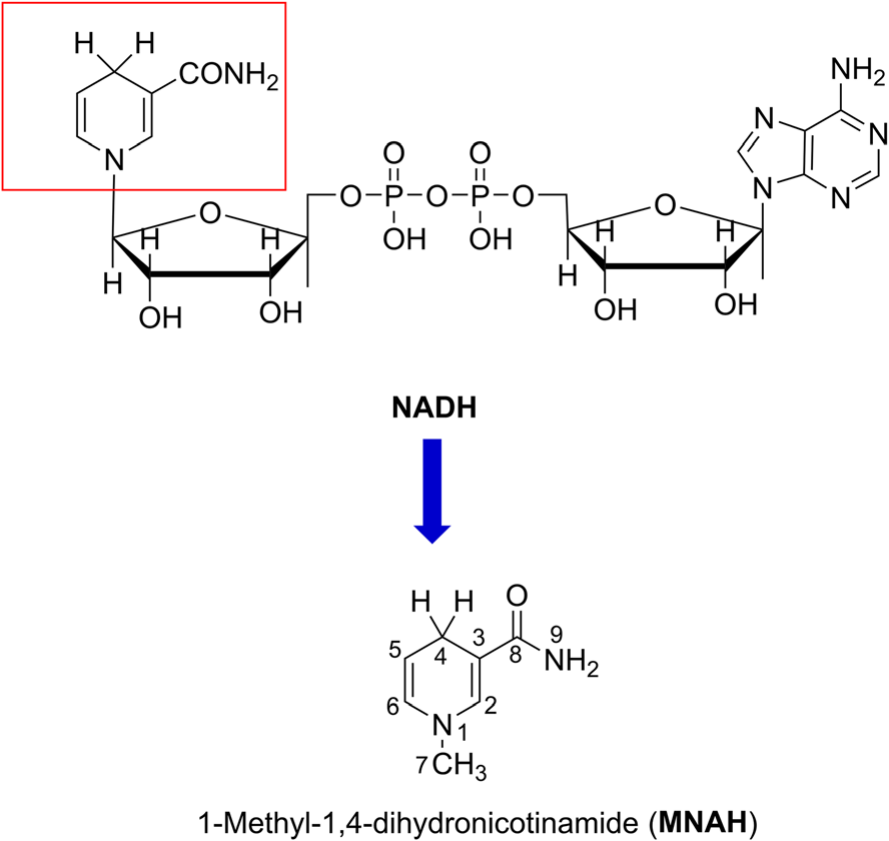
The structure of MNAH.

Since the active center of NADH is dihydronicotinamide (Fig. 1), which contains two weak C4–H bonds; the radical scavenging could occur directly there following the formal hydrogen transfer mechanism.^13^ The nicotinamide component may also react with highly-reactive ROS, such as HO˙ radicals, through the radical adduct formation (RAF) and either the hydrogen transfer pathway or single electron transfer (SET). Nevertheless, this matter has not yet been thoroughly investigated.

The hydroxyl radical is a prevalent and highly reactive species among free radicals. It is identified as the primary effector of tissue damage caused by ionizing radiation and oxidative damage to DNA.^14,15^ Because of its high reactivity its physiological lifetime is short, therefore the ideal way of reducing oxidative stress due to HO˙ would be to inhibit the production of hydroxyl radicals.^16^ Due to its dominant role in pathologic processes it is quite common in the literature to investigate radical scavenging activity against the hydroxyl radical, and it is indeed crucial if the focus is on evaluating the degradation of organic compounds.^17–19^ On the other hand, the HO˙ model may not be an effective way to compare the radical scavenging activity of organic compounds due to the inherent high reactivity of this radical. A more representative model of the typical less reactive radicals is the HOO˙ radical, and thus it is a better alternative for computational studies to evaluate the yet unknown free radical scavenging activity of compounds.^16,17,19^ To highlight this issue in this study we examine and compare activity against HO˙ and HOO˙.

Previous studies demonstrated that the HO˙/HOO˙ radical scavenging activity of organic compounds can be accurately modeled by quantum chemical methods.^20–22^ Using this method, we modeled the kinetics and mechanism of the HO˙/HOO˙ scavenging activity of 1-methyl-1,4-dihydronicotinamide (MNAH) (Fig. 1), the active center of NADH, in physiological environments.

## 2. Computational methods

The kinetic calculations in this study were conducted in accordance with the quantum mechanics-based test for overall free radical scavenging activity (QM-ORSA) protocol, combined with the SMD solvation model^23^ procedure for pentyl ethanoate and water solvents.^17,24–27^ The traditional transition state theory (TST) at a temperature of 298.15 K and a standard state of 1 M was used to compute the rate constant (*k*) as outlined in eqn (1) by using the Eyringpy code.^26^ (more information method in Table S1, ESI†):^28–33^1
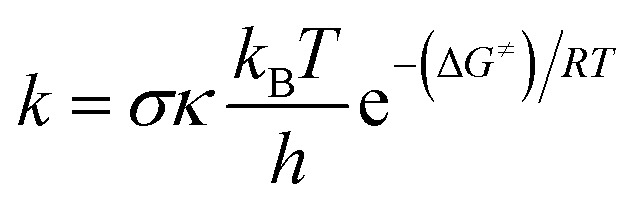
where *σ* is the reaction symmetry number,^34,35^*κ* stands for tunneling corrections that were calculated using Eckart barrier,^36^*k*_B_ is the Boltzmann constant, *h* is the Planck constant, Δ*G*^≠^ is Gibbs free energy of activation.

Gaussian 16 software^37^ was employed to conduct all calculations at the M06-2X/6-311++G(d,p) level of theory, which was previously identified as an appropriate model chemistry for this application.^38,39^

## 3. Results and discussions

### 3.1. The radical scavenging in the gas phase

#### 3.1.1. Thermodynamic study

Under conditions of non-polar media such as in the gas phase, the antiradical activity can follow either of three primary mechanisms: sequential electron transfer proton transfer (SETPT),^40,41^ formal hydrogen transfer (FHT),^25^ or radical adduct formation (RAF) in the case of molecules with double bonds.^42^ To identify the most effective antioxidant mechanisms, we calculated the Gibbs free energy changes (Δ*G*°) in the gas phase for each reaction of MNAH with HOO˙ and HO˙ radicals in one of the following reactions: FHT, RAF, or single electron transfer (SET) for the SETPT reaction. The results are shown in Table 1.

**Table 1:** The computed BDE and Δ*G*° (in kcal mol^−1^) following the RAF, FHT, and SET mechanisms of the MNAH + HO˙/HOO˙ reactions

Mechanisms	Positions	BDE	Δ*G*°	
			HO˙	HOO˙
FHT	C4–H	71.2	−45.8	−14.6
	C7–H	90.9	−26.1	5.1
	N9–H	109.9	−6.2	25.1
RAF	C2		−23.1	
	C3		−17.4	
	C5		−25.7	
	C6		−20.2	
SET			139.6	144.3

The findings revealed that most reactions between HO˙ and MNAH were thermodynamically favorable (Δ*G*° < 0), except for the SET reaction (Δ*G*° = 139.6 kcal mol^−1^). The MNAH + HOO˙ reaction was only spontaneous at the FHT (C4–H, Δ*G*° = −14.6 kcal mol^−1^), whereas those of other mechanisms, such as the SET and FHT (C7–H and N9–H), are not thermodynamically spontaneous (Δ*G*° = 5.1–144.3 kcal mol^−1^). The H-abstraction of C4–H is the most preferred thermodynamically MNAH + HO˙ reaction (Δ*G*° = −45.8 kcal mol^−1^, BDE = 71.2 kcal mol^−1^). Thus, this could make a significant contribution to the HO˙ radical scavenging activity of MNAH. Nevertheless, the MNAH + HO˙ reaction could also follow the RAF reactions at C2, C3, C5, and C6 and the FHT (C7–H) due to the low negative Δ*G*° values (Δ*G*° = −17.4 to −26.1 kcal mol^−1^). The HO˙/HOO˙ radical scavenging activity of MNAH may not involve the H-abstraction of N9–H due to the high BDE and Δ*G*° values (BDE = 109.9 kcal mol; Δ*G*° = −6.2 and 25.1 kcal mol^−1^ for HO˙ and HOO˙ radicals, respectively). Consequently, the kinetics of the HO˙/HOO˙ radicals scavenging activity of MNAH were evaluated at all of the sites of spontaneous reactions (Δ*G*° < 0).

#### 3.1.2. Kinetic study

**Fig. 2:**
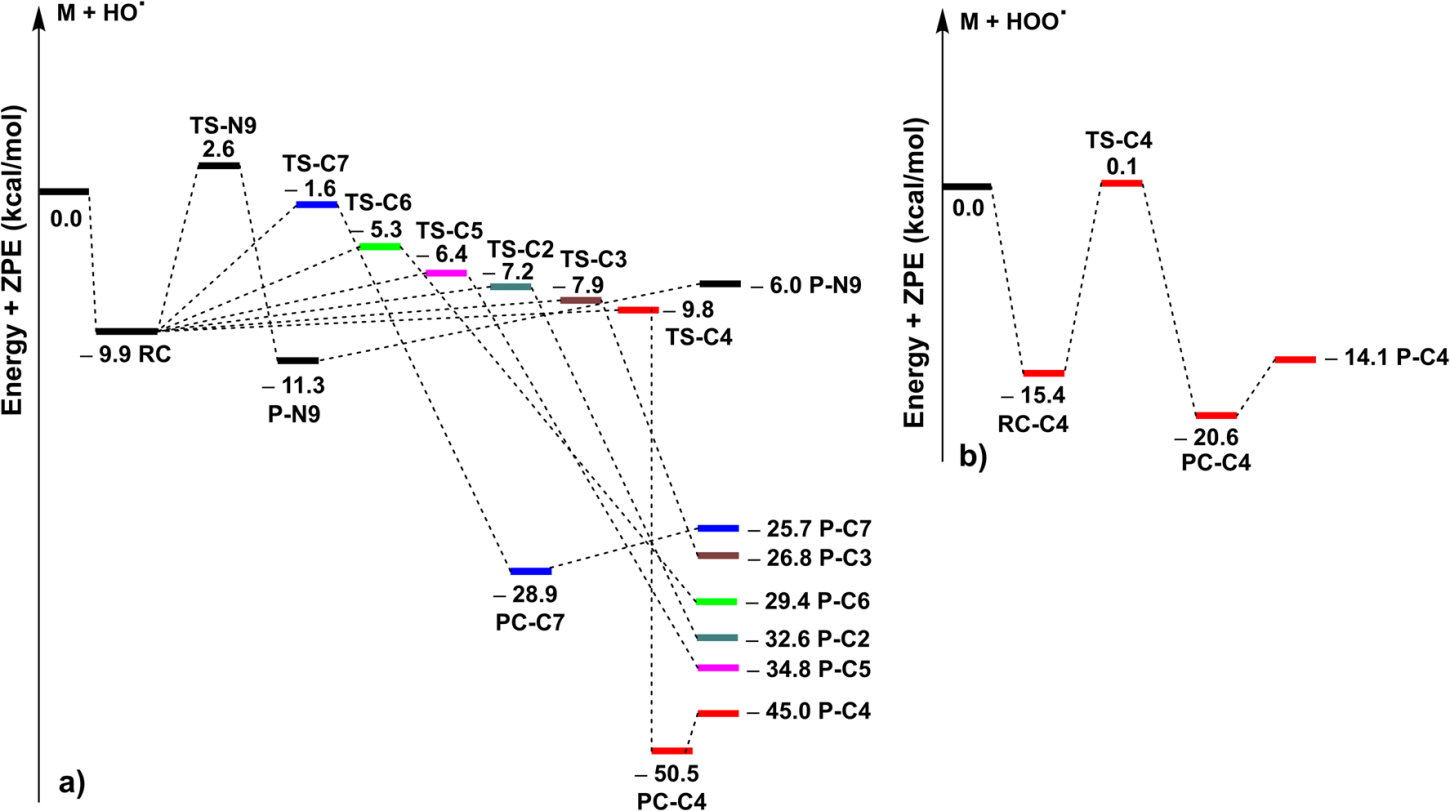
The PES of the MNAH + HO˙ (a)/HOO˙ (b) reactions at the spontaneous reactions (RC: pre-complex; TS: transition state; PC: post-complex; P: product).

In the initial kinetic evaluation, the potential energy surfaces (PES) were first computed; the findings are presented in Fig. 2. The highest reaction barrier (12.5 kcal mol^−1^) for the MNAH + HO˙ reaction is observed at the FHT of the N9–H bond, while the C4 position of MNAH presented the lowest reaction barrier value (0.1 kcal mol^−1^). The RAF at C2, C3, and C5 positions had reaction barriers of 2.7, 2.0, and 3.5 kcal mol^−1^, respectively, whereas those of C6 and C7–H were 4.6 and 8.3 kcal mol^−1^, respectively. Based on these results, the dominant MNAH + HO˙ reactions are the addition of the HO˙ radical at the C2, C3, or C5 positions and the FHT reaction of the C4–H bonds, while the H-abstraction of MNAH by HO˙ radicals *via* the C7–H and N9–H bonds would not contribute to the activity. The HOO˙ radical scavenging reaction is defined by the H-abstraction at the C4–H bond with the low reaction barrier at 0.1 kcal mol^−1^.

**Fig. 3:**
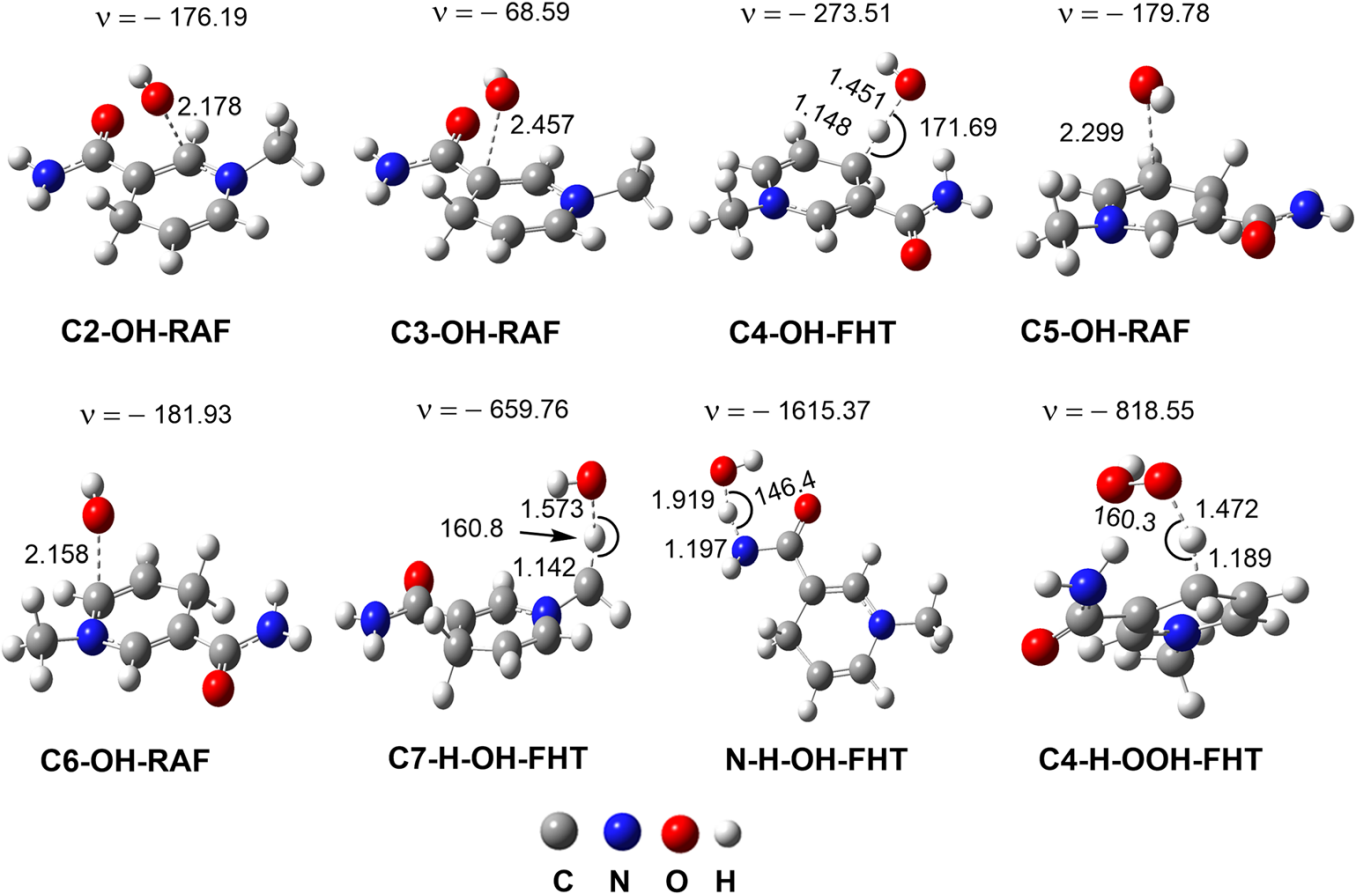
The optimized transition states of the RAF and FHT mechanisms in the MNAH + HO˙/HOO˙ reactions (*ν* in cm^−1^, bond length in Å).

The kinetics of the MNAH + HO˙/HOO˙ reactions were calculated by using the QM-ORSA methodology.^17^ The results are presented in Table 2, whereas the optimized structures and the SOMO orbitals of transition states (TS) are shown in Fig. 3 and S1, ESI,† respectively. In the gas phase, the FHT reaction of the C4–H with HO˙ radicals was barrierless (Δ*G*^≠^ ≈ 0 kcal mol^−1^, *k*_Eck_ = 6.02 × 10^12^ M^−1^ s^−1^, *Γ* = 27.1%), whereas that of C7–H and N9–H bonds had no contribution to the radical scavenging activity with *k*_Eck_ = 1.39 × 10^10^ M^−1^ s^−1^ (*Γ* = 0.1%) and 5.60 × 10^7^ M^−1^ s^−1^ (*Γ* = 0.0%), respectively. At the same time, the RAF reactions at C2, C3, and C5 form a substantial part of the overall MNAH + HO˙ reaction with Δ*G*^≠^ ≈ 0 kcal mol^−1^, *k*_Eck_ = 6.02 × 10^12^ M^−1^ s^−1^, *Γ* = 27.1% for each position. The addition reaction at the C6 location contributed only about 1.1% to the overall rate constant. Thus in the gas phase, the MNAH + HO˙ reaction was rapid and defined by the FHT(C4–H) and RAF(C2, C3, and C5) mechanisms with the overall rate constant *k*_overall_ = 2.22 × 10^13^ M^−1^ s^−1^, whereas the MNAH + HOO˙ reaction was moderate and characterized by the FHT(C4–H) with *k*_overall_ = 2.83 × 10^6^ M^−1^ s^−1^. The main products of the MNAH + HO˙ reaction in the gas phase were P-C2 (27.1%), P-C3 (27.1%), P-C4 (27.1%), and P-C5 (17.6%), whereas for the MNAH + HOO˙ reaction P-C4(HOO) was the only product (100%) (Fig. 2 and Table 2).

**Table 2:** Computed Δ*G*^≠^ (in kcal mol^−1^), *κ*, *k*_Eck_ (M^−1^ s^−1^) and branching ratios (*Γ*%) for the MNAH + HO˙/HOO˙ reactions^a^

Radicals	Mechanisms	Positions	Δ*G*^≠^	*κ*	*k*_Eck_	*Γ*
HO˙	FHT	C4–H	0.0	1.0	6.02 × 10^12^	27.1
		C7–H	3.9	1.6	1.39 × 10^10^	0.1
		N9–H	8.9	30.2	5.60 × 10^7^	0.0
	RAF	C2	0.0	1.0	6.02 × 10^12^	27.1
		C3	0.0	1.0	6.02 × 10^12^	27.1
		C5	0.3	1.0	3.91 × 10^12^	17.6
		C6	2.0	1.0	2.35 × 10^11^	1.1
	***k***_**overall**_				**2.22** × **10**^**13**^	
HOO˙	FHT	C4–H	9.1	2.1	2.83 × 10^6^	100.0

a *Γ* = *k*_Eck_·100/*k*_overall_.

The extremely high theoretical rate constant for the MNAH + HO˙ reaction (*k*_overall_ = 2.22 × 10^13^ M^−1^ s^−1^) suggests that the reaction is diffusion-limited even in the gas phase where the collision rate at the given temperature would limit the reaction to 10^9^–10^10^ M^−1^ s^−1^. Hence the activity against HO˙ radical is not a useful basis for comparison. On the other hand, the HOO˙ radical scavenging activity of MNAH is comparable to the reference antioxidant Trolox (*k*_(HOO˙ + Trolox)_ = 1.87 × 10^7^ M^−1^ s^−1^).^33^ This suggests that MNAH may exhibit a good radical scavenging activity in the physiological environment that warrants further investigation.

### 3.2. The HO˙/HOO˙ scavenging activity of MNAH in the physiological environments

#### 3.2.1. Acid–base equilibrium of MNAH in water

**Fig. 4:**
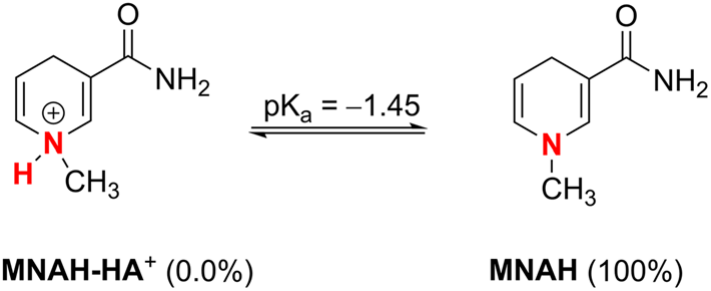
Dissociation equilibria of MNAH at pH = 7.4.

Sequential proton loss electron transfer (SPLET) is the principal antioxidant mechanism that controls the radical scavenging efficacy of nitrogenous substances in aqueous solutions.^43^ This is mainly attributable to the spontaneous deprotonation, which removes the potential barrier of the initial phase. Therefore, this section evaluated the deprotonation equilibria of MNAH. The p*K*_a_ value was determined using a literature method,^44^ as illustrated in Fig. 4. It was determined that the p*K*_a_ value of MNAH in an aqueous solution was −1.45 (N1–H). MNAH molecule is exclusively present in a neutral state (MNAH, 100%) in the physiological aqueous solution. Thus, the neutral state was assessed in the HO˙/HOO˙ scavenging activity of MNAH in the physiological environments (water and pentyl ethanoate).

#### 3.2.2. Kinetic study

The HO˙/HOO˙ antiradical activity of MNAH in physiological media was calculated using the QM-ORSA protocol.^17,19^ The overall rate constants of the HO˙/HOO˙ + MNAH reaction were calculated using eqn (2)–(5). The findings are presented in Table 3.

**Table 3:** The computed Δ*G*^≠^ (in kcal mol^−1^), branching ratios (*Γ*%), and *k*_app_, *k*_f_, *k*_overall_, (M^−1^ s^−1^) of the reaction between MNAH and HO˙/HOO˙ in the physiological environment

Radicals	Mechanism		Pentyl ethanoate				Water				
			Δ*G*^≠^	*κ*	*k*_app_	*Γ*	Δ*G*^≠^	*κ*	*k*_app_	*k*_f_	*Γ*
HO˙	SET						2.5	4.6^a^	7.40 × 10^9^	7.40 × 10^9^	26.7
	FHT	C4–H	∼0	1.0	3.20 × 10^9^	18.9	∼0	1.0	3.10 × 10^9^	3.10 × 10^9^	11.2
		C7–H	4.4	1.8	2.20 × 10^9^	13.0	∼0	1.0	3.10 × 10^9^	3.10 × 10^9^	11.2
		N9–H	12.0	37.6	3.80 × 10^5^	0.0	∼0	1.0	2.90 × 10^9^	2.90 × 10^9^	10.5
	RAF	C2	1.7	1.0	2.60 × 10^9^	15.4	∼0	1.0	2.60 × 10^9^	2.60 × 10^9^	9.4
		C3	1.5	1.0	3.20 × 10^9^	18.9	∼0	1.0	3.00 × 10^9^	3.00 × 10^9^	10.8
		C5	1.0	1.0	3.10 × 10^9^	18.3	∼0	1.0	3.00 × 10^9^	3.00 × 10^9^	10.8
		C6	2.0	1.0	2.60 × 10^9^	15.4	∼0	1.0	2.60 × 10^9^	2.60 × 10^9^	9.4
	***k***_**overall**_				**1.69** × **10**^**10**^					**2.77** × **10**^**10**^	
HOO˙	SET						11.1	16.6^a^	4.30 × 10^4^	4.30 × 10^4^	1.8
	FHT	C4–H	11.9	1.6	2.00 × 10^4^		9.3	2.4	2.40 × 10^6^	2.40 × 10^6^	98.2
***k***_**overall**_					**2.00** × **10**^**4**^					**2.44** × **10**^**6**^	

a The nuclear reorganization energy (*λ*, in kcal mol^−1^).

In pentyl ethanoate:

2 *k*_overall_(HO˙) = Σ*k*_app_(RAF) + Σ*k*_app_(FHT)

3 *k*_overall_(HOO˙) = *k*_app_(FHT(C4–H))

In water:

4 *k*_overall_(HO˙) = *k*_f_(SET) + Σ*k*_f_(RAF) + Σ*k*_f_(FHT)

5 *k*_overall_(HOO˙) = *k*_f_(FHT) + *k*_f_(SET)

The *k*_overall_ values for the MNAH + HO˙ reaction in the nonpolar and aqueous environments were 1.69 × 10^10^ and 2.77 × 10^10^ M^−1^ s^−1^, respectively. The *k*_overall_ of the MNAH + HOO˙ reaction is slower compared to the hydroxyl radical, with values of 2.00 × 10^4^ and 2.44 × 10^6^ M^−1^ s^−1^ in the pentyl ethanoate and water solvents, respectively. It is important to notice that the p*K*_a_ value of the HOO˙ radical is 4.88. Consequently, the molar fraction of HOO˙ is 0.137 at pH = 5.6, resulting in a *k*_overall_(MNAH + HOO˙) of 3.84 × 10^5^ M^−1^ s^−1^. This is in excellent agreement with the experimental data (*k*_exp_(NADH + HOO˙) = (1.8 ± 0.2)×10^5^ M^−1^ s^−1^).^45^ It was found that the HO˙ scavenging activity of MNAH was defined by the FHT of C4–H and C7–H positions (31.9%) and RAF (68.0%) reactions in the nonpolar environment. Conversely, the H-abstraction of N9–H bond did not contribute to the MNAH + HO˙ reaction. On the other hand, the H-abstraction of the C4–H bond dominated the activity against HOO˙ (100%).

The HO˙ antiradical activity in the polar environment is a combination of all analyzed mechanisms (FHT (32.9%), SET (26.7%) and RAF (40.4%)). Formal hydrogen transfer was the driving force behind the activity against the HOO˙ radical, where the SET reaction contributed only 1.8% of the overall rate constant. Based on the findings, the FHT and RAF reactions with HO˙ radicals in the aqueous physiological environment were barrierless (Δ*G*^≠^ ≈ 0 kcal mol^−1^). Consequently, the *k*_app_ values of these processes were diffusion-limited (cannot exceed diffusion rates *k*_D_) and accounted for approximately 73.3% of the overall rate constant.

According to the results the MNAH + HO˙ reaction is practically diffusion-limited under all conditions, including the gas phase. On the other hand, the activity against HOO˙ was more nuanced. MNAH exhibits a higher HOO˙ antiradical activity in the lipid medium than *trans*-resveratrol (∼1.5 times, *k* = 1.31 × 10^4^ M^−1^ s^−1^)^46^ and ascorbic acid (∼3.5 times, *k* = 5.71 × 10^3^ M^−1^ s^−1^),^17^ but it is inferior to Trolox (∼5.0 times, *k* = 1.00 × 10^5^ M^−1^ s^−1^).^33^ In the polar medium MNAH exhibits a higher activity than Trolox (∼18.5 times, *k* = 1.30 × 10^5^ M^−1^ s^−1^),^33^ but it is weaker than ascorbic acid and *trans*-resveratrol. The markedly different activity against the two radicals is arguably the result of the high reactivity of HO˙ and not the exceptional specific activity of MNAH in targeting and eliminating HO˙. Thus our results underscore the importance of comparing antioxidant activity against the less reactive free radicals that have longer lifetimes under physiological conditions. Nevertheless, our results suggest that MNAH is an efficient radical scavenger in key physiological environments.

## 4. Conclusions

The radical scavenging activity of MNAH against HO˙ and HOO˙ was assessed through density functional theory calculations. In the lipid and water media, the *k*_overall_ values of the HO˙ + MNAH reaction were 1.69 × 10^10^ and 2.77 × 10^10^ M^−1^ s^−1^, respectively. The HOO˙ radical scavenging activity was measured at 2.00 × 10^4^ and 2.44 × 10^6^ M^−1^ s^−1^. In water, at pH = 5.6, the calculated rate constant (*k*_overall_(MNAH + HOO˙) = 3.84 × 10^5^ M^−1^ s^−1^) is in good agreement with the experimental data (*k*_exp_(NADH + HOO˙) = (1.8 ± 0.2)×10^5^ M^−1^ s^−1^) and could verify the accuracy of the computing method. In both nonpolar and polar media, the HOO˙ antiradical activity of MNAH was defined by the H-abstraction of the C4–H bond, whereas the HO˙ antiradical activity was determined by a combination of the SET (in polar media), RAF, and FHT reactions. The hydroperoxyl radical scavenging activity of MNAH is greater than that of *trans*-resveratrol and ascorbic acid in the lipid medium, and Trolox in the aqueous physiological environment. The results have verified the potent radical scavenger role of MNAH in physiological environments, also highlighting that HOO˙ is a better model for comparing antiradical activity than the highly reactive HO˙.

## Data availability

The data supporting this article have been included as part of the ESI.†

## Conflicts of interest

There are no conflicts to declare.

## Supplementary Material

RA-014-D4RA07184K-s001
