# Supplementary material for: The radical scavenging activity of 1-methyl-1,4-dihydronicotinamide: theoretical insights into the mechanism, kinetics and solvent effects
Source: RSC Adv. 2024 Nov 20;14(50):37196–201. doi: 10.1039/d4ra07184k (PMC11578042; doi:10.1039/d4ra07184k)
Supplement: RA-014-D4RA07184K-s001 [file RA-014-D4RA07184K-s001.pdf]

Supplementary Information (SI) for RSC Advances.
This journal is © The Royal Society of Chemistry 2024

Supporting Information (SI)

The radical scavenging activity of 1-methyl-1,4-dihydronicotinamide: Theoretical insights into the mechanism, kinetics and solvent effects

Quan V. Vo[1*], Nguyen Thi Hoa[1], and Adam Mechler[2*]

[1]*The University of Danang - University of Technology and Education, Danang 550000, Vietnam.*
[2]*Department of Biochemistry and Chemistry, La Trobe University, Victoria 3086, Australia.*
*Corresponding author: vvquan@ute.udn.vn; a.mechler@latrobe.edu.au

Table of Contents

Table S1. The method to calculate rate constant following the conventional transition state theory S2

Figure S1. The SOMO orbitals of the transition states following the RAF and FHT mechanisms in the MNAH + $HO^{\bullet}$/$HOO^{\bullet}$ reactions S3

Table S2: The Cartesian coordinates and energies of TS of the reaction between MNAH with $HO^{\bullet}$/$HOO^{\bullet}$ following the FHT and FHT mechanisms (G: the gas phase; P: pentyl ethanoate; W: water) S4

References S15

Table S1. The method to calculate rate constant following the conventional transition state theory

The rate constant (k) was calculated by using the conventional transition state theory (TST) (at 298.15 K, 1M standard state) according to the equation (1):[1-5]

$$k = \sigma\kappa \frac{k_B T}{h} e^{-(\Delta G^{\neq})/RT} \qquad (1)$$

Where: σ is the reaction symmetry number ,[6-7]
κ contains the tunneling corrections calculated using the Eckart barrier,[8]
k_B is the Boltzmann constant,
h is the Planck constant,
$\Delta G^{\neq}$ is the Gibbs free energy of activation.

The Marcus Theory was used to estimate the reaction barriers of SET reactions.[9-12] The free energy of reaction $\Delta G^{\neq}$ for the SET pathway was computed following the equations (2,3).

$$\Delta G^{\neq}_{SET} = \frac{\lambda}{4}\left(1 + \frac{\Delta G^{0}_{SET}}{\lambda}\right)^2 \qquad (2)$$

$$\lambda \approx \Delta E_{SET} - \Delta G^{0}_{SET} \qquad (3)$$

where ΔG_{SET} is the Gibbs energy of reaction, ΔE_{SET} is the non-adiabatic energy difference between reactants and vertical products for SET.[13-14]

For rate constants that were close to the diffusion limit a correction was applied to yield realistic results[15]. The apparent rate constants (k_{app}) were calculated following the Collins–Kimball theory in the solvents at 298.15K;[16] the steady-state Smoluchowski rate constant (k_D) for an irreversible bimolecular diffusion–controlled reaction was calculated following the literature as corroding to equations (4,5).[15,17]

$$k_{app} = \frac{k_{TST} k_D}{k_{TST} + k_D} \qquad (4)$$

$$k_D = 4\pi R_{AB} D_{AB} N_A \qquad (5)$$

where R_{AB} is the reaction distance, N_A is the Avogadro constant, and $D_{AB} = D_A + D_B$ (D_{AB} is the mutual diffusion coefficient of the reactants A and B),[16,18] where D_A or D_B is estimated using the Stokes–Einstein formulation (6).[19-20]

$$D_{A\ or\ B} = \frac{k_B T}{6\pi\eta a_{A\ or\ B}} \qquad (6)$$

η is the viscosity of the solvents (i.e. $\eta(H_2O) = 8.91\times10^{-4}$ Pa s, η(pentyl ethanoate) = 8.62×10^{-4} Pa s) and a is the radius of the solute.

The kinetic study requires different considerations. Water (dielectric constants, $\varepsilon = 78.35$) and pentyl ethanoate ($\varepsilon = 4.73$) are the *de facto* standard solvents in the literature to mimic the polar and nonpolar environments in the human body.[15,21-23] Thus, these solvents were used to model the physiological environments. The solvent cage effects were included following the corrections proposed by Okuno,[24] adjusted with the free volume theory according to the Benson correction[15,25-27] to reduce over-penalizing entropy losses in solution. For the species that have multiple conformers, all of these were investigated and the conformer with the lowest electronic energy was included in the analysis.[22-23] The hindered internal rotation treatment was also applied to the single bonds to ensure that the obtained conformer has the lowest electronic energy.[23,28] All transition states were characterized by the existence of only one single imaginary frequency. Intrinsic coordinate calculations (IRCs) were performed to ensure that each transition state is connected correctly with the pre-complex and post-complex.

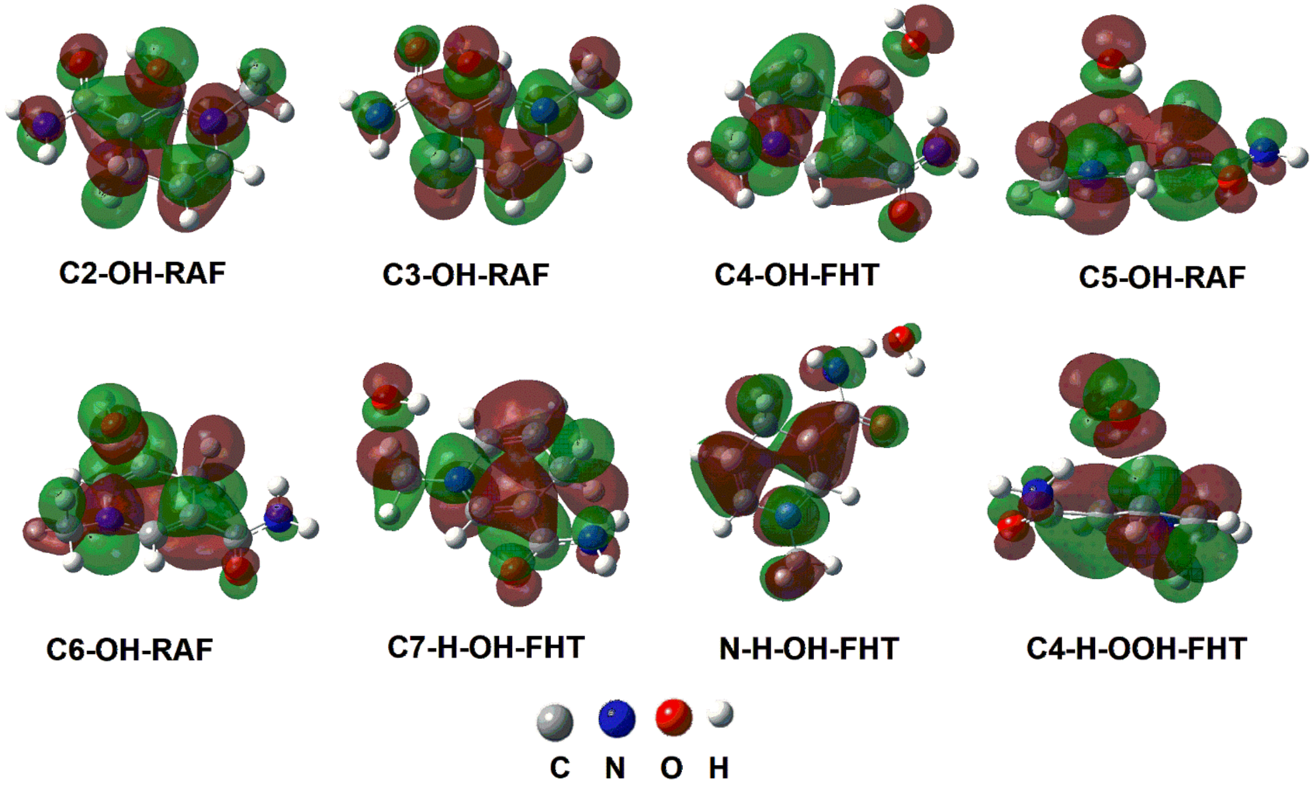

Figure S1. The SOMO orbitals of the transition states following the RAF and FHT mechanisms in the MNAH + HO•/HOO• reactions

Table S2: The Cartesian coordinates and energies of TS of the reaction between MNAH with HO•/HOO• following the FHT and FHT mechanisms (G: the gas phase; P: pentyl ethanoate; W: water)

**Name**	**C2-OH-RAF-G**
Cartesian Coordinates	Frequency and Energy
O 2.13692100 -1.62515900 -0.02642600	Zero-point correction= 0.179200 (Hartree/Particle)
N -1.81627600 -0.55708500 -0.30959300	Thermal correction to Energy= 0.190867
N 2.85107400 0.51750200 0.05031800	Thermal correction to Enthalpy= 0.191811
C 0.16219500 1.54214700 0.10133500	Thermal correction to Gibbs Free Energy= 0.141054
C 0.48362200 0.07954500 -0.04239100	Sum of electronic and zero-point Energies= -532.977866
C -0.50965800 -0.86544500 -0.16505000	Sum of electronic and thermal Energies= -532.966199
C -1.32058600 1.77504100 -0.00358000	Sum of electronic and thermal Enthalpies= -532.965255
C -2.19457600 0.78728300 -0.17573600	Sum of electronic and thermal Free Energies= -533.016012
C -2.81288400 -1.58763500 -0.06070600	
C 1.87134600 -0.43329900 -0.00533300	
H 0.68310700 2.12786500 -0.66913200	
H 0.52363200 1.90580500 1.07385700	
H -0.23439900 -1.90765100 -0.26789600	
H -1.69164900 2.78957600 0.06388900	
H -3.26057100 0.96286300 -0.24421500	
H -3.74149900 -1.32278500 -0.56651600	
H -2.99173600 -1.69722700 1.01263500	
H -2.45502900 -2.53513500 -0.46216600	
H 3.79318700 0.19012300 0.19391000	
H 2.64564300 1.48067100 0.25250900	
O -0.22547900 -0.78687500 1.99321300	
H 0.52654900 -1.39649400 2.04087800	
**Name**	**C3-OH-RAF-G**
Cartesian Coordinates	Frequency and Energy
O 2.27227600 -1.51221300 0.19873800	Zero-point correction= 0.178700 (Hartree/Particle)
N -1.70736600 -0.53670900 -0.21386900	Thermal correction to Energy= 0.190680
N 2.94067600 0.61399900 -0.15165400	Thermal correction to Enthalpy= 0.191624
C 0.24568300 1.61557300 0.00215600	Thermal correction to Gibbs Free Energy= 0.139829
C 0.58148100 0.14438700 -0.04583000	Sum of electronic and zero-point Energies= -532.979068
C -0.37914800 -0.80808300 -0.15279200	Sum of electronic and thermal Energies= -532.967088
C -1.24810400 1.80417500 0.05577100	Sum of electronic and thermal Enthalpies= -532.966144
C -2.10945400 0.79374800 -0.04364700	Sum of electronic and thermal Free Energies= -533.017939
C -2.68052100 -1.59684100 -0.01025200	
C 1.98041300 -0.33894800 0.00894800	
H 0.65185500 2.14335500 -0.87420300	
H 0.70776500 2.07181700 0.88717500	
H -0.09066700 -1.85259400 -0.18826300	
H -1.64034000 2.80590400 0.17600800	
H -3.18133900 0.94103600 -0.00430600	
H -3.59980900 -1.35493900 -0.54473400	
H -2.90324400 -1.73068600 1.05280400	
H -2.28346100 -2.52862900 -0.41090500	
H 3.90124600 0.33260000 -0.04063200	
H 2.72422800 1.59146600 -0.23674200	
O 0.29735200 -0.42616100 2.32741900	
H 0.95761300 -1.11786900 2.14893400	
**Name**	**C5-OH-RAF-G**
Cartesian Coordinates	Frequency and Energy
O 2.26964500 -1.52589700 0.12671500	Zero-point correction= 0.178658 (Hartree/Particle)

N -1.72369800 -0.52366800 -0.18820300<br>N 2.93211900 0.63114700 0.10176400<br>C 0.24414900 1.61743600 -0.11300700<br>C 0.57465200 0.14396300 -0.03002200<br>C -0.38022700 -0.80223100 -0.08531600<br>C -1.24101500 1.81559800 0.01834300<br>C -2.11888700 0.79355200 -0.05999700<br>C -2.70140400 -1.58268100 0.01498100<br>C 1.97619800 -0.34548000 0.06576100<br>H 0.60612200 2.04671000 -1.05940300<br>H 0.73440100 2.17529200 0.69453600<br>H -0.10026500 -1.84886600 -0.04983200<br>H -1.62750300 2.81908500 0.12269500<br>H -3.18568500 0.95497500 0.02112400<br>H -3.62134200 -1.33037200 -0.51208700<br>H -2.92353300 -1.72155100 1.07742600<br>H -2.31152200 -2.51277500 -0.39605300<br>H 3.89396400 0.33199200 0.08295400<br>H 2.72521800 1.59695800 -0.08412500<br>O -1.34664100 1.57458900 2.30224200<br>H -0.74244500 0.81475700 2.28051100	Thermal correction to Energy= 0.190634<br>Thermal correction to Enthalpy= 0.191578<br>Thermal correction to Gibbs Free Energy= 0.139786<br>Sum of electronic and zero-point Energies= -532.976702<br>Sum of electronic and thermal Energies= -532.964726<br>Sum of electronic and thermal Enthalpies= -532.963782<br>Sum of electronic and thermal Free Energies= -533.015574
**Name**	**C6-OH-RAF-G**
Cartesian Coordinates	Frequency and Energy
O 2.28505000 -1.49566700 -0.31522000<br>N -1.75017800 -0.58652500 -0.25529900<br>N 2.88925500 0.64325200 0.08469700<br>C 0.18032800 1.57700000 0.03070600<br>C 0.54238100 0.11730800 -0.10392800<br>C -0.39427400 -0.83706800 -0.20121400<br>C -1.30115400 1.76498300 -0.08998700<br>C -2.16869300 0.71405200 -0.14076600<br>C -2.68937100 -1.64440800 0.08795900<br>C 1.95516500 -0.34099600 -0.11692000<br>H 0.69383500 2.17517400 -0.73374000<br>H 0.50327200 1.97199600 1.00588900<br>H -0.09588300 -1.87661400 -0.27112100<br>H -1.70780500 2.76791100 -0.10438900<br>H -3.23565800 0.86750800 -0.23615900<br>H -3.64505200 -1.45134500 -0.40074800<br>H -2.83721700 -1.68518400 1.17020100<br>H -2.30091500 -2.59645000 -0.27133500<br>H 3.84099200 0.33383900 0.20571600<br>H 2.63522200 1.53526000 0.47406600<br>O -2.07376900 0.89808300 2.00739700<br>H -2.59119000 1.68781100 2.21840100	Zero-point correction= 0.178904 (Hartree/Particle)<br>Thermal correction to Energy= 0.190629<br>Thermal correction to Enthalpy= 0.191573<br>Thermal correction to Gibbs Free Energy= 0.140836<br>Sum of electronic and zero-point Energies= -532.974830<br>Sum of electronic and thermal Energies= -532.963105<br>Sum of electronic and thermal Enthalpies= -532.962161<br>Sum of electronic and thermal Free Energies= -533.012899
**Name**	**C4-OH-FHT-G**
Cartesian Coordinates	Frequency and Energy
O 2.34281200 -1.44949200 -0.32946400<br>N -1.70843500 -0.54556500 -0.04992300<br>N 2.87423600 0.73564600 0.00046600<br>C 0.24924400 1.57506400 -0.18248600<br>C 0.57846700 0.13161300 -0.10339600<br>C -0.37011700 -0.82450600 -0.03738100<br>C -1.22326900 1.79564900 -0.18283200	Zero-point correction= 0.176165 (Hartree/Particle)<br>Thermal correction to Energy= 0.187810<br>Thermal correction to Enthalpy= 0.188754<br>Thermal correction to Gibbs Free Energy= 0.137548<br>Sum of electronic and zero-point Energies= -532.982112<br>Sum of electronic and thermal Energies= -532.970467<br>Sum of electronic and thermal Enthalpies= -532.969523

C	-2.10027900	0.78246800	-0.10647300	Sum of electronic and thermal Free Energies= -533.020729
C	-2.69258900	-1.58477600	0.20206800	
C	1.99721900	-0.29535200	-0.14455800	
H	0.72390900	2.03955800	-1.06224000	
H	0.74078000	2.12098700	0.70049700	
H	-0.08104200	-1.86858100	0.00098700	
H	-1.60771700	2.80500000	-0.25735300	
H	-3.17085400	0.94564200	-0.11048100	
H	-3.59434000	-1.38078000	-0.37615500	
H	-2.95190500	-1.64231500	1.26292800	
H	-2.28776600	-2.54391500	-0.11727100	
H	3.84511000	0.46679800	0.04313000	
H	2.61061500	1.58012400	0.50009300	
O	1.41335700	2.95904200	1.67623400	
H	0.67324600	3.57859300	1.75251600	
**Name**				**C7-OH-FHT-G**
Cartesian Coordinates				Frequency and Energy
O	2.11830900	-1.64186400	0.17012600	Zero-point correction= 0.175022 (Hartree/Particle)
N	-1.81715400	-0.51452100	-0.35827900	Thermal correction to Energy= 0.187361
N	2.83083000	0.49278700	0.33735200	Thermal correction to Enthalpy= 0.188305
C	0.18306100	1.56264400	0.05357100	Thermal correction to Gibbs Free Energy= 0.134088
C	0.47594100	0.07969500	0.01725000	Sum of electronic and zero-point Energies= -532.968900
C	-0.49189200	-0.83276300	-0.17821400	Sum of electronic and thermal Energies= -532.956562
C	-1.30445100	1.79979400	0.01473600	Sum of electronic and thermal Enthalpies= -532.955617
C	-2.18910900	0.82202100	-0.18339200	Sum of electronic and thermal Free Energies= -533.009835
C	-2.82480700	-1.52827400	-0.25295600	
C	1.85553800	-0.45309400	0.16983100	
H	0.66219400	2.07728300	-0.79317000	
H	0.60505400	2.01676200	0.95979000	
H	-0.23701300	-1.88613900	-0.20501600	
H	-1.67345200	2.81007100	0.13768700	
H	-3.25637700	1.00213600	-0.21279700	
H	-3.70525200	-1.26032500	-0.83740100	
H	-3.17888200	-1.63683200	0.82754200	
H	-2.43139100	-2.49050200	-0.57621600	
H	3.78371500	0.16585800	0.35606800	
H	2.66405900	1.47170200	0.18232800	
O	-3.45505000	-1.30961400	2.34169900	
H	-2.60809100	-0.86972100	2.52482800	
**Name**				**N9-OH-FHT-G**
Cartesian Coordinates				Frequency and Energy
O	2.46637700	-1.16633000	-0.36151300	Zero-point correction= 0.174066 (Hartree/Particle)
N	-1.56935600	-0.56301800	0.18513400	Thermal correction to Energy= 0.185742
N	2.88827100	1.06430600	-0.56889300	Thermal correction to Enthalpy= 0.186686
C	0.15342600	1.77118300	-0.13335200	Thermal correction to Gibbs Free Energy= 0.134552
C	0.62483500	0.33074800	-0.14739700	Sum of electronic and zero-point Energies= -532.962331
C	-0.23023900	-0.70653400	0.00102900	Sum of electronic and thermal Energies= -532.950655
C	-1.34957700	1.81865900	-0.00890400	Sum of electronic and thermal Enthalpies= -532.949710
C	-2.10285300	0.72982000	0.13994000	Sum of electronic and thermal Free Energies= -533.001845
C	-2.46653600	-1.70414500	0.21409300	
C	2.03997500	-0.02183300	-0.33528700	
H	0.47118300	2.29290900	-1.04425200	
H	0.60469500	2.32742500	0.70217100	
H	0.15543000	-1.71962000	-0.02116500	

H -1.84101900 2.78301100 -0.03401200<br>H -3.18050200 0.77968700 0.23461800<br>H -3.00016400 -1.81632400 -0.73453500<br>H -3.19348200 -1.58693800 1.01989100<br>H -1.88773000 -2.60708300 0.40097200<br>H 4.04307500 0.74966200 -0.55096000<br>H 2.71253600 1.86734500 0.03120500<br>O 4.91334700 0.20148100 0.05050500<br>H 4.51813500 -0.68750000 0.09664000	
**Name**	**C2-OH-RAF-P**
Cartesian Coordinates	Frequency and Energy
O 2.14483300 -1.62604200 -0.05222900<br>N -1.81620800 -0.56125300 -0.23514200<br>N 2.84447000 0.52122000 0.05049200<br>C 0.16529900 1.53839400 0.09539700<br>C 0.48530700 0.07399800 -0.01026000<br>C -0.52023100 -0.87816400 -0.09209100<br>C -1.31755100 1.77364700 0.01986800<br>C -2.19756100 0.78583100 -0.12704900<br>C -2.84038700 -1.58845800 -0.11003800<br>C 1.87493100 -0.42899600 -0.00520000<br>H 0.66883200 2.09769200 -0.70541600<br>H 0.55841500 1.94033500 1.03964700<br>H -0.25838900 -1.92342000 -0.19211100<br>H -1.68342700 2.79109000 0.08019900<br>H -3.26523700 0.95613700 -0.18509700<br>H -3.68986000 -1.33240100 -0.74360500<br>H -3.17281800 -1.67672100 0.92797100<br>H -2.43010300 -2.54261200 -0.43756900<br>H 3.80213100 0.21539800 0.14121300<br>H 2.63964600 1.49220600 0.22484700<br>O -0.15988700 -0.78684600 2.03665800<br>H 0.59073000 -1.39740300 2.06331900	Zero-point correction= 0.178935 (Hartree/Particle)<br>Thermal correction to Energy= 0.190558<br>Thermal correction to Enthalpy= 0.191502<br>Thermal correction to Gibbs Free Energy= 0.141080<br>Sum of electronic and zero-point Energies= -532.998854<br>Sum of electronic and thermal Energies= -532.987231<br>Sum of electronic and thermal Enthalpies= -532.986287<br>Sum of electronic and thermal Free Energies= -533.036709
**Name**	**C3-OH-RAF-P**
Cartesian Coordinates	Frequency and Energy
O 2.28186200 -1.45766700 0.38954900<br>N -1.70351300 -0.52148300 -0.17338900<br>N 2.93032400 0.63833300 -0.14007100<br>C 0.24039900 1.63768400 -0.02431800<br>C 0.58122500 0.16505500 0.00567700<br>C -0.37945700 -0.79304500 -0.08682000<br>C -1.25247400 1.82210100 0.07228100<br>C -2.11332100 0.80739700 -0.01085800<br>C -2.69181700 -1.58393500 -0.05954100<br>C 1.97997100 -0.29921500 0.09219900<br>H 0.61231400 2.11263300 -0.94505500<br>H 0.73713000 2.15841800 0.80333500<br>H -0.10339600 -1.84112200 -0.10098200<br>H -1.64471800 2.82399900 0.19658200<br>H -3.18600400 0.94665800 0.04459500<br>H -3.55481200 -1.34755900 -0.68296700<br>H -3.01835900 -1.70725800 0.97750800<br>H -2.25338600 -2.51754300 -0.40908700<br>H 3.90005800 0.36174800 -0.09810000	Zero-point correction= 0.178834 (Hartree/Particle)<br>Thermal correction to Energy= 0.190497<br>Thermal correction to Enthalpy= 0.191442<br>Thermal correction to Gibbs Free Energy= 0.140684<br>Sum of electronic and zero-point Energies= -532.998833<br>Sum of electronic and thermal Energies= -532.987169<br>Sum of electronic and thermal Enthalpies= -532.986225<br>Sum of electronic and thermal Free Energies= -533.036982

H 2.70877000 1.56706600 -0.46196200<br>O 0.33488300 -0.67814800 2.46934400<br>H 1.03145400 -1.23973100 2.08220400	
**Name**	**C5-OH-RAF-P**
Cartesian Coordinates	Frequency and Energy
O 2.27847200 -1.50877800 0.20627100<br>N -1.71358200 -0.49950000 -0.18822000<br>N 2.94274700 0.64195100 0.05732400<br>C 0.26409500 1.63517700 -0.14204600<br>C 0.58708900 0.15965900 -0.02722100<br>C -0.37791100 -0.78209900 -0.07408600<br>C -1.22310200 1.84342600 -0.04847500<br>C -2.10043400 0.82687100 -0.06715300<br>C -2.70306800 -1.54293500 0.04704200<br>C 1.98468000 -0.32556300 0.08377200<br>H 0.64277200 2.04838200 -1.08861800<br>H 0.76140700 2.20784000 0.65167000<br>H -0.11369200 -1.83185200 -0.01841900<br>H -1.60343200 2.84974600 0.07020000<br>H -3.16829100 0.98997900 0.00489900<br>H -3.60766400 -1.31637200 -0.51745600<br>H -2.95157400 -1.61954800 1.11019200<br>H -2.30539700 -2.49566300 -0.29972200<br>H 3.90874300 0.34959900 0.06242400<br>H 2.74044800 1.60374900 -0.16325400<br>O -1.55230800 1.21055300 2.42388700<br>H -0.75769800 0.67791100 2.24799700	Zero-point correction= 0.177835 (Hartree/Particle)<br>Thermal correction to Energy= 0.190036<br>Thermal correction to Enthalpy= 0.190980<br>Thermal correction to Gibbs Free Energy= 0.138081<br>Sum of electronic and zero-point Energies= -532.998001<br>Sum of electronic and thermal Energies= -532.985800<br>Sum of electronic and thermal Enthalpies= -532.984856<br>Sum of electronic and thermal Free Energies= -533.037755
**Name**	**C6-OH-RAF-P**
Cartesian Coordinates	Frequency and Energy
O 2.29331400 -1.52025400 -0.20286300<br>N -1.74505500 -0.59124600 -0.19065300<br>N 2.89030000 0.63929900 0.06289200<br>C 0.18946700 1.56800400 0.03639900<br>C 0.54904600 0.10413300 -0.06115400<br>C -0.39403200 -0.84979400 -0.14353300<br>C -1.29259500 1.75712300 -0.04313100<br>C -2.16640500 0.70597800 -0.07807700<br>C -2.69920000 -1.67071700 0.02139100<br>C 1.96226900 -0.34811900 -0.06900700<br>H 0.67414100 2.14137000 -0.76571000<br>H 0.55544900 1.99986800 0.97940500<br>H -0.11207000 -1.89453000 -0.19836700<br>H -1.69652100 2.76115100 -0.05591200<br>H -3.23324900 0.85912600 -0.17112200<br>H -3.64300200 -1.41525800 -0.46070400<br>H -2.87046100 -1.83275900 1.08881900<br>H -2.31135300 -2.58386200 -0.42876600<br>H 3.85862900 0.36762600 0.14917000<br>H 2.64300300 1.58410100 0.31000900<br>O -2.08317000 0.98678900 2.06964900<br>H -2.63416400 1.77189100 2.19546800	Zero-point correction= 0.178077 (Hartree/Particle)<br>Thermal correction to Energy= 0.190043<br>Thermal correction to Enthalpy= 0.190988<br>Thermal correction to Gibbs Free Energy= 0.139394<br>Sum of electronic and zero-point Energies= -532.997432<br>Sum of electronic and thermal Energies= -532.985466<br>Sum of electronic and thermal Enthalpies= -532.984521<br>Sum of electronic and thermal Free Energies= -533.036115
**Name**	**C4-OH-FHT-P**
Cartesian Coordinates	Frequency and Energy
O 2.29181300 -1.45858400 -0.22026100	Zero-point correction= 0.175118 (Hartree/Particle)

N -1.72424900 -0.56442700 -0.04714000	Thermal correction to Energy= 0.186761
N 2.87433600 0.71439300 0.07132900	Thermal correction to Enthalpy= 0.187705
C 0.17492800 1.56989300 -0.16162900	Thermal correction to Gibbs Free Energy= 0.136183
C 0.54741500 0.14995300 -0.06840600	Sum of electronic and zero-point Energies= -533.012263
C -0.38999200 -0.83099800 -0.02627600	Sum of electronic and thermal Energies= -533.000621
C -1.27711700 1.77482000 -0.09700000	Sum of electronic and thermal Enthalpies= -532.999677
C -2.14924800 0.74742400 -0.04283500	Sum of electronic and thermal Free Energies= -533.051199
C -2.69785000 -1.64183000 0.09509000	
C 1.97823000 -0.28521000 -0.06716900	
H 0.62382200 2.04529700 -1.04715200	
H 0.70923900 2.20765700 0.71800200	
H -0.09977000 -1.87379600 0.00794300	
H -1.67152400 2.78367100 -0.09406300	
H -3.22185900 0.88784000 -0.00711600	
H -3.58162500 -1.40812500 -0.49761600	
H -2.98204100 -1.76055600 1.14315700	
H -2.25764200 -2.56779100 -0.26973200	
H 3.84289300 0.43445000 0.13227500	
H 2.61980800 1.62543500 0.46166400	
O 1.53574300 2.99053600 1.63122700	
H 1.11537200 3.84084900 1.46461100	
**Name**	**C7-OH-FHT-P**
Cartesian Coordinates	Frequency and Energy
O 2.11883000 -1.64853200 0.13889500	Zero-point correction= 0.174446 (Hartree/Particle)
N -1.82715300 -0.50745200 -0.34610600	Thermal correction to Energy= 0.186931
N 2.81983300 0.48752000 0.31852800	Thermal correction to Enthalpy= 0.187875
C 0.18503600 1.56123600 0.04731800	Thermal correction to Gibbs Free Energy= 0.133142
C 0.47055800 0.07564900 0.01358700	Sum of electronic and zero-point Energies= -532.991048
C -0.50614900 -0.83363200 -0.17087500	Sum of electronic and thermal Energies= -532.978562
C -1.29820200 1.81057200 -0.03009500	Sum of electronic and thermal Enthalpies= -532.977618
C -2.19133900 0.83695600 -0.21554700	Sum of electronic and thermal Free Energies= -533.032351
C -2.84163500 -1.51608700 -0.25074000	
C 1.85091600 -0.45245400 0.15642800	
H 0.69145600 2.07442300 -0.78279300	
H 0.58645700 2.01265500 0.96426400	
H -0.27008600 -1.89128900 -0.19480700	
H -1.65691500 2.82933200 0.05213500	
H -3.25608100 1.02588000 -0.27907500	
H -3.72494200 -1.22733600 -0.82071900	
H -3.17684500 -1.65198700 0.82372200	
H -2.45975400 -2.47426900 -0.60011500	
H 3.77796600 0.17981700 0.39294400	
H 2.63583100 1.47704800 0.28924800	
O -3.36625200 -1.41134600 2.45038400	
H -2.55575100 -0.87960100 2.53878600	
**Name**	**N9-OH-FHT-P**
Cartesian Coordinates	Frequency and Energy
O 2.48812300 -1.17752200 -0.30517100	Zero-point correction= 0.174010 (Hartree/Particle)
N -1.57534100 -0.56930600 0.04133400	Thermal correction to Energy= 0.185602
N 2.89408500 1.04718300 -0.56230000	Thermal correction to Enthalpy= 0.186546
C 0.15801300 1.76131600 -0.20707300	Thermal correction to Gibbs Free Energy= 0.135012
C 0.63208400 0.31997700 -0.18684400	Sum of electronic and zero-point Energies= -532.981284
C -0.24048600 -0.71960700 -0.08439300	Sum of electronic and thermal Energies= -532.969692
C -1.33363200 1.81675100 0.00890600	Sum of electronic and thermal Enthalpies= -532.968747

C -2.09740100 0.72955700 0.11289600<br>C -2.46832900 -1.70276000 0.22929500<br>C 2.04339700 -0.03247300 -0.32062400<br>H 0.41290700 2.24751400 -1.15718100<br>H 0.65825100 2.35326800 0.57225000<br>H 0.13011100 -1.73816700 -0.09573800<br>H -1.80992400 2.78765700 0.07126700<br>H -3.16933900 0.77799600 0.25909500<br>H -3.30375700 -1.63713100 -0.47016100<br>H -2.85628800 -1.72464500 1.25102300<br>H -1.91960700 -2.62286800 0.03627300<br>H 4.02216300 0.75681200 -0.47103100<br>H 2.67332400 1.89100800 -0.03541600<br>O 4.91697400 0.22814200 0.18808900<br>H 4.51449700 -0.65579000 0.26143100	Sum of electronic and thermal Free Energies= -533.020282
**Name**	**C2-OH-RAF-W**
Cartesian Coordinates	Frequency and Energy
O 2.14498700 -1.61920100 -0.05038600<br>N -1.80390700 -0.55768400 -0.22098600<br>N 2.84306700 0.52423200 0.09555200<br>C 0.16187000 1.54090100 0.05986800<br>C 0.48972500 0.08383500 -0.00713900<br>C -0.52483100 -0.88240800 -0.06739300<br>C -1.31840400 1.77659200 0.05094600<br>C -2.19984400 0.79014200 -0.08772500<br>C -2.84100600 -1.58465500 -0.15017100<br>C 1.88078000 -0.40955800 0.00923400<br>H 0.62602500 2.06810500 -0.78609100<br>H 0.60804400 1.98884000 0.95830700<br>H -0.27127100 -1.92981700 -0.15664200<br>H -1.67923300 2.79289600 0.14301100<br>H -3.26992600 0.94698500 -0.11436400<br>H -3.64015900 -1.33758800 -0.84750800<br>H -3.24482200 -1.63580000 0.86334800<br>H -2.40726900 -2.54516800 -0.41937600<br>H 3.80834400 0.22578300 0.11434900<br>H 2.64095400 1.51040300 0.16302700<br>O -0.16569700 -0.84844200 2.11219300<br>H 0.58550700 -1.45476100 2.12175100	Zero-point correction= 0.178389 (Hartree/Particle)<br>Thermal correction to Energy= 0.190134<br>Thermal correction to Enthalpy= 0.191078<br>Thermal correction to Gibbs Free Energy= 0.140340<br>Sum of electronic and zero-point Energies= -533.012533<br>Sum of electronic and thermal Energies= -533.000788<br>Sum of electronic and thermal Enthalpies= -532.999844<br>Sum of electronic and thermal Free Energies= -533.050582
**Name**	**C3-OH-RAF-W**
Cartesian Coordinates	Frequency and Energy
O 2.25930000 -1.55345600 0.00478400<br>N -1.70360700 -0.55414200 -0.07398300<br>N 2.92304900 0.60154200 -0.12275000<br>C 0.21056100 1.56007300 -0.01282900<br>C 0.56955100 0.11594700 -0.00553700<br>C -0.40440400 -0.85748500 -0.10455200<br>C -1.25452800 1.77497400 0.14190200<br>C -2.13080000 0.76547300 0.09919900<br>C -2.72685400 -1.59881000 -0.15251100<br>C 1.98562400 -0.35047200 -0.03503200<br>H 0.54488800 2.02131600 -0.95773300<br>H 0.75469600 2.09752000 0.77242600<br>H -0.14667800 -1.90550400 -0.18062800	Zero-point correction= 0.177611 (Hartree/Particle)<br>Thermal correction to Energy= 0.189618<br>Thermal correction to Enthalpy= 0.190563<br>Thermal correction to Gibbs Free Energy= 0.138739<br>Sum of electronic and zero-point Energies= -533.016600<br>Sum of electronic and thermal Energies= -533.004592<br>Sum of electronic and thermal Enthalpies= -533.003648<br>Sum of electronic and thermal Free Energies= -533.055472

H -1.62210100 2.78505900 0.26813000<br>H -3.20111600 0.89000900 0.18875900<br>H -3.40451300 -1.36731000 -0.97375800<br>H -3.28362600 -1.61872100 0.78472800<br>H -2.24290300 -2.55629400 -0.32311600<br>H 3.89546300 0.32502500 -0.12326700<br>H 2.70252800 1.58618000 -0.13554500<br>O 0.73187900 -0.10321600 2.55603600<br>H 0.98072600 -1.00331900 2.32540100	
**Name**	**C5-OH-RAF-W**
Cartesian Coordinates	Frequency and Energy
O 2.27460500 -1.54195600 0.26238100<br>N -1.72975500 -0.52531700 -0.05673100<br>N 2.92656000 0.60059900 -0.00727400<br>C 0.22348400 1.56166500 -0.08716800<br>C 0.57180600 0.10996400 0.02875800<br>C -0.38206200 -0.83662500 0.02940000<br>C -1.23900800 1.79210800 0.01304900<br>C -2.14031600 0.75709300 -0.04974900<br>C -2.68856900 -1.63212600 -0.10169300<br>C 1.98912400 -0.35084400 0.10597300<br>H 0.57791200 1.96119500 -1.05187700<br>H 0.73708500 2.15780000 0.67608000<br>H -0.15411700 -1.89276100 0.08805200<br>H -1.61385500 2.80467600 -0.00677200<br>H -3.20894100 0.92335700 -0.06221800<br>H -3.69286300 -1.22597400 -0.18277700<br>H -2.59611900 -2.22176600 0.81043600<br>H -2.46775300 -2.25848500 -0.96562300<br>H 3.89953200 0.32830200 0.02996000<br>H 2.70554400 1.57483400 -0.15106200<br>O -1.27908200 1.92808100 2.54806500<br>H -0.78091000 1.10871300 2.46179800	Zero-point correction= 0.178389 (Hartree/Particle)<br>Thermal correction to Energy= 0.190251<br>Thermal correction to Enthalpy= 0.191196<br>Thermal correction to Gibbs Free Energy= 0.139485<br>Sum of electronic and zero-point Energies= -533.014826<br>Sum of electronic and thermal Energies= -533.002964<br>Sum of electronic and thermal Enthalpies= -533.002019<br>Sum of electronic and thermal Free Energies= -533.053730
**Name**	**C6-OH-RAF-W**
Cartesian Coordinates	Frequency and Energy
O 2.28328000 -1.53719800 0.03063600<br>N -1.74645300 -0.57694600 -0.20617900<br>N 2.88539900 0.63215300 0.10099900<br>C 0.18697800 1.56636500 -0.01759000<br>C 0.54715200 0.10326300 -0.02287800<br>C -0.39414000 -0.85045800 -0.11569700<br>C -1.28911000 1.75968500 -0.01175900<br>C -2.16976900 0.70122000 -0.04558200<br>C -2.70029300 -1.67571200 -0.06112500<br>C 1.95931200 -0.34067200 0.03831200<br>H 0.61322300 2.07623200 -0.89392500<br>H 0.61919300 2.07882900 0.85275200<br>H -0.13471400 -1.90131800 -0.13659200<br>H -1.69275600 2.76196900 0.03038600<br>H -3.23880900 0.85776100 -0.09163300<br>H -3.66033400 -1.36668100 -0.47005600<br>H -2.81918100 -1.93817000 0.99247100<br>H -2.33530600 -2.53946100 -0.61377500<br>H 3.86040000 0.37054900 0.14550000	Zero-point correction= 0.178363 (Hartree/Particle)<br>Thermal correction to Energy= 0.190132<br>Thermal correction to Enthalpy= 0.191077<br>Thermal correction to Gibbs Free Energy= 0.140230<br>Sum of electronic and zero-point Energies= -533.011586<br>Sum of electronic and thermal Energies= -532.999817<br>Sum of electronic and thermal Enthalpies= -532.998872<br>Sum of electronic and thermal Free Energies= -533.049719

H 2.64886000 1.61263800 0.10998700	
O -2.06535900 0.98057000 2.18257900	
H -2.62323000 1.76530400 2.24737200	
**Name**	**C4-OH-FHT-W**
Cartesian Coordinates	Frequency and Energy
O 2.34391400 -1.37666700 -0.33115900	Zero-point correction= 0.175098 (Hartree/Particle)
N -1.73099600 -0.56199600 -0.05379100	Thermal correction to Energy= 0.186794
N 2.81418000 0.79635500 0.14195900	Thermal correction to Enthalpy= 0.187739
C 0.15727700 1.57903600 -0.19714000	Thermal correction to Gibbs Free Energy= 0.135269
C 0.53333200 0.15533400 -0.11316000	Sum of electronic and zero-point Energies= -533.023636
C -0.39274500 -0.83001400 -0.04646200	Sum of electronic and thermal Energies= -533.011940
C -1.29939300 1.77944200 -0.18108000	Sum of electronic and thermal Enthalpies= -533.010996
C -2.16421800 0.74575700 -0.10030600	Sum of electronic and thermal Free Energies= -533.063466
C -2.69741300 -1.63509000 0.15851900	
C 1.97259300 -0.22850600 -0.10603700	
H 0.61789200 2.05646400 -1.07741400	
H 0.70994400 2.13183400 0.65686800	
H -0.10387900 -1.87235700 0.00340100	
H -1.70126100 2.78240700 -0.24905800	
H -3.23821100 0.87866600 -0.09330100	
H -3.59686900 -1.42531400 -0.41852700	
H -2.95180100 -1.70884600 1.21789000	
H -2.26364000 -2.57395800 -0.17879400	
H 3.79221700 0.55507800 0.21954200	
H 2.49171400 1.63598500 0.62830000	
O 1.74087600 2.84159600 1.57257900	
H 1.22717100 3.65569400 1.62607600	
**Name**	**C7-OH-FHT-W**
Cartesian Coordinates	Frequency and Energy
O 2.08189700 -1.60250500 0.29131100	Zero-point correction= 0.173804 (Hartree/Particle)
N -1.82448900 -0.39016800 -0.22696900	Thermal correction to Energy= 0.185423
N 2.85253000 0.51739600 0.28436600	Thermal correction to Enthalpy= 0.186367
C 0.22320400 1.61403500 -0.08927700	Thermal correction to Gibbs Free Energy= 0.134854
C 0.48763300 0.14789100 0.01845900	Sum of electronic and zero-point Energies= -533.011956
C -0.51294000 -0.75695600 -0.05068800	Sum of electronic and thermal Energies= -533.000337
C -1.22354900 1.91325800 -0.25267800	Sum of electronic and thermal Enthalpies= -532.999393
C -2.15988300 0.95260200 -0.31076000	Sum of electronic and thermal Free Energies= -533.050906
C -2.85185800 -1.37524700 -0.14249000	
C 1.86914400 -0.38957500 0.20775600	
H 0.78239500 2.04543400 -0.93352700	
H 0.60615500 2.14227000 0.79666900	
H -0.33189000 -1.82063700 0.02867900	
H -1.53299900 2.94744700 -0.33153900	
H -3.21528900 1.15508500 -0.43038100	
H -3.73808700 -1.04138800 -0.67892000	
H -3.13905200 -1.50375700 1.01948100	
H -2.48738600 -2.33353800 -0.50795400	
H 3.80162500 0.19213300 0.40958500	
H 2.69110400 1.51078900 0.20949700	
O -3.45413700 -1.64016900 2.47830200	
H -2.91834700 -2.40729600 2.70644300	
**Name**	**N9-OH-FHT-W**
Cartesian Coordinates	Frequency and Energy
O 2.34304200 -1.28420700 -0.03202700	Zero-point correction= 0.172224 (Hartree/Particle)

N -1.67604000 -0.52292400 0.04219200<br>N 2.91068400 0.89251400 -0.27994300<br>C 0.09417200 1.69492400 0.02253800<br>C 0.56142400 0.28677900 -0.03247700<br>C -0.33376700 -0.73851300 -0.02484400<br>C -1.37336200 1.82375700 0.15344500<br>C -2.18874400 0.74777300 0.15170900<br>C -2.59538000 -1.66216500 0.13020700<br>C 2.02380100 -0.07185600 -0.12108900<br>H 0.41780600 2.23709400 -0.88445200<br>H 0.59044100 2.24517400 0.83756600<br>H -0.01914400 -1.77123500 -0.07669600<br>H -1.80633100 2.81130700 0.24482000<br>H -3.26538600 0.81191200 0.23203600<br>H -2.14214500 -2.51927700 -0.35952500<br>H -3.53008000 -1.39774800 -0.36000700<br>H -2.78099500 -1.88489600 1.18201700<br>H 4.12715400 0.59339000 -0.27864800<br>H 2.53650400 1.82829300 -0.35954100<br>O 5.31965900 0.32702700 -0.18523600<br>H 5.55651500 -0.31021400 -0.86611500	Thermal correction to Energy= 0.183663<br>Thermal correction to Enthalpy= 0.184608<br>Thermal correction to Gibbs Free Energy= 0.132404<br>Sum of electronic and zero-point Energies= -533.032619<br>Sum of electronic and thermal Energies= -533.021179<br>Sum of electronic and thermal Enthalpies= -533.020235<br>Sum of electronic and thermal Free Energies= -533.072439
**Name**	**C4-OOH-FHT-G**
Cartesian Coordinates	Frequency and Energy
O 2.17832300 -1.36602600 0.03304700<br>N -1.81796800 -0.34222800 0.11237900<br>N 2.82827600 0.80857300 0.12678700<br>C 0.14303800 1.70419700 -0.36088300<br>C 0.47888800 0.29407100 -0.05223500<br>C -0.48358200 -0.62986200 0.17312200<br>C -1.31112800 1.89740000 -0.55502000<br>C -2.20233900 0.92757200 -0.29686000<br>C -2.81674400 -1.39363400 0.22036300<br>C 1.89033800 -0.18307900 0.05349800<br>H 0.75288000 2.12949300 -1.16473000<br>H 0.50239200 2.35495400 0.56819500<br>H -0.20839400 -1.65219300 0.40410700<br>H -1.67918800 2.85985500 -0.88658200<br>H -3.26957300 1.07076900 -0.41015200<br>H -3.13428900 -1.74109600 -0.76703100<br>H -3.68509200 -1.02101800 0.76493800<br>H -2.39396200 -2.23147200 0.77187200<br>H 3.76000800 0.48125900 0.33663400<br>H 2.56429600 1.69141100 0.55023100<br>H 0.27112500 1.58469100 3.02029000<br>O 1.15794500 2.71856700 1.83529100<br>O 1.09994400 1.53543700 2.52466800	Zero-point correction= 0.181100 (Hartree/Particle)<br>Thermal correction to Energy= 0.193663<br>Thermal correction to Enthalpy= 0.194607<br>Thermal correction to Gibbs Free Energy= 0.140999<br>Sum of electronic and zero-point Energies= -608.124680<br>Sum of electronic and thermal Energies= -608.112117<br>Sum of electronic and thermal Enthalpies= -608.111173<br>Sum of electronic and thermal Free Energies= -608.164781
**Name**	**C4-OOH-FHT-P**
Cartesian Coordinates	Frequency and Energy
O 2.21034900 -1.36149000 0.07260500<br>N -1.80341000 -0.35202600 0.06774300<br>N 2.82837100 0.82033800 0.13324900<br>C 0.16123200 1.69818100 -0.37077100<br>C 0.49264300 0.28452200 -0.05941700<br>C -0.47921500 -0.63987900 0.15651800	Zero-point correction= 0.180996 (Hartree/Particle)<br>Thermal correction to Energy= 0.193619<br>Thermal correction to Enthalpy= 0.194563<br>Thermal correction to Gibbs Free Energy= 0.140842<br>Sum of electronic and zero-point Energies= -608.148734<br>Sum of electronic and thermal Energies= -608.136111

C -1.29249900 1.89007400 -0.58333700<br>C -2.18643200 0.91901600 -0.33856000<br>C -2.82375500 -1.37560700 0.25604200<br>C 1.90009100 -0.17726300 0.06808000<br>H 0.77141500 2.11822900 -1.17819700<br>H 0.50031200 2.34802000 0.54559700<br>H -0.22100600 -1.66244300 0.40419200<br>H -1.65522200 2.85287600 -0.92130700<br>H -3.25302800 1.05818900 -0.46495800<br>H -3.31523900 -1.60291600 -0.69285000<br>H -3.56848400 -1.02921200 0.97431300<br>H -2.35342500 -2.27827500 0.64047800<br>H 3.76743400 0.52579500 0.36517900<br>H 2.54806800 1.72232400 0.50095000<br>H 0.20154900 1.49647700 2.97133000<br>O 1.11916100 2.72231300 1.90120000<br>O 1.07628300 1.52040200 2.55385000	Sum of electronic and thermal Enthalpies= -608.135167<br>Sum of electronic and thermal Free Energies= -608.188888
**Name**	**C4-OOH-FHT-W**
Cartesian Coordinates	Frequency and Energy
O 2.22301300 -1.31563000 0.35581200<br>N -1.77431100 -0.31410100 0.09524500<br>N 2.86273300 0.82373800 0.02935100<br>C 0.19108500 1.72485200 -0.35327700<br>C 0.52100500 0.31353400 -0.02691100<br>C -0.46005700 -0.60454600 0.20934300<br>C -1.25788200 1.90904300 -0.61030000<br>C -2.15515100 0.94517500 -0.35924800<br>C -2.80604200 -1.32609500 0.30847600<br>C 1.91753700 -0.13103700 0.14251900<br>H 0.81022000 2.14656000 -1.15216900<br>H 0.48428400 2.36132600 0.57143600<br>H -0.21876100 -1.62033600 0.49664000<br>H -1.61060400 2.86200600 -0.98374300<br>H -3.21908300 1.07295400 -0.51269200<br>H -3.30473400 -1.55684900 -0.63441800<br>H -3.54098900 -0.95529000 1.02320100<br>H -2.34151800 -2.22629900 0.70345600<br>H 3.82082900 0.56931300 0.22593500<br>H 2.62539300 1.80495200 0.02274900<br>H 0.04250000 1.05700200 2.75567200<br>O 0.86157800 2.62136400 2.12338900<br>O 0.95414900 1.33600800 2.57146200	Zero-point correction= 0.180404 (Hartree/Particle)<br>Thermal correction to Energy= 0.193445<br>Thermal correction to Enthalpy= 0.194389<br>Thermal correction to Gibbs Free Energy= 0.139329<br>Sum of electronic and zero-point Energies= -608.156805<br>Sum of electronic and thermal Energies= -608.143764<br>Sum of electronic and thermal Enthalpies= -608.142820<br>Sum of electronic and thermal Free Energies= -608.197880

References

(1) Evans, M. G.; Polanyi, M. Some Applications of the Transition State Method to the Calculation of Reaction Velocities, Especially in Solution. *Trans. Faraday Soc.* **1935**, *31*, 875-894.

(2) Eyring, H. The Activated Complex in Chemical Reactions. *J. Chem. Phys.* **1935**, *3*, 107-115.

(3) Truhlar, D. G.; Hase, W. L.; Hynes, J. T. Current Status of Transition-State Theory. *J. Phys. Chem.* **1983**, *87*, 2664-2682.

(4) Furuncuoglu, T.; Ugur, I.; Degirmenci, I.; Aviyente, V. Role of Chain Transfer Agents in Free Radical Polymerization Kinetics. *Macromolecules* **2010**, *43*, 1823-1835.

(5) Vélez, E.; Quijano, J.; Notario, R.; Pabón, E.; Murillo, J.; Leal, J.; Zapata, E.; Alarcón, G. A Computational Study of Stereospecifity in the Thermal Elimination Reaction of Menthyl Benzoate in the Gas Phase. *J. Phys. Org. Chem.* **2009**, *22*, 971-977.

(6) Pollak, E.; Pechukas, P. Symmetry Numbers, Not Statistical Factors, Should Be Used in Absolute Rate Theory and in Broensted Relations. *J. Am. Chem. Soc.* **1978**, *100*, 2984-2991.

(7) Fernández-Ramos, A.; Ellingson, B. A.; Meana-Pañeda, R.; Marques, J. M.; Truhlar, D. G. Symmetry Numbers and Chemical Reaction Rates. *Theor. Chem. Acc.* **2007**, *118*, 813-826.

(8) Eckart, C. The Penetration of a Potential Barrier by Electrons. *Phy. Rev.* **1930**, *35*, 1303.

(9) Marcus, R. A. Chemical and Electrochemical Electron-Transfer Theory. *Annu. Rev. Phys. Chem.* **1964**, *15*, 155-196.

(10) Marcus, R. A. Electron Transfer Reactions in Chemistry. Theory and Experiment. *Rev. Mod. Phys.* **1993**, *65*, 599.

(11) Lu, Y.; Wang, A.; Shi, P.; Zhang, H. A Theoretical Study on the Antioxidant Activity of Piceatannol and Isorhapontigenin Scavenging Nitric Oxide and Nitrogen Dioxide Radicals. *PloS one* **2017**, *12*, e0169773.

(12) Lu, Y.; Wang, A.; Shi, P.; Zhang, H.; Li, Z. Quantum Chemical Study on the Antioxidation Mechanism of Piceatannol and Isorhapontigenin toward Hydroxyl and Hydroperoxyl Radicals. *PloS one* **2015**, *10*, e0133259.

(13) Nelsen, S. F.; Blackstock, S. C.; Kim, Y. Estimation of Inner Shell Marcus Terms for Amino Nitrogen Compounds by Molecular Orbital Calculations. *J. Am. Chem. Soc.* **1987**, *109*, 677-682.

(14) Nelsen, S. F.; Weaver, M. N.; Luo, Y.; Pladziewicz, J. R.; Ausman, L. K.; Jentzsch, T. L.; O'Konek, J. J. Estimation of Electronic Coupling for Intermolecular Electron Transfer from Cross-Reaction Data. *J. Phys. Chem. A* **2006**, *110*, 11665-11676.

(15) Galano, A.; Alvarez-Idaboy, J. R. A Computational Methodology for Accurate Predictions of Rate Constants in Solution: Application to the Assessment of Primary Antioxidant Activity. *J. Comput. Chem.* **2013**, *34*, 2430-2445.

(16) Collins, F. C.; Kimball, G. E. Diffusion-Controlled Reaction Rates. *J. Colloid Sci.* **1949**, *4*, 425-437.

(17) Von Smoluchowski, M. Mathematical Theory of the Kinetics of the Coagulation of Colloidal Solutions. *Z. Phys. Chem* **1917**, *92*, 129-68.

(18) Truhlar, D. G. Nearly Encounter-Controlled Reactions: The Equivalence of the Steady-State and Diffusional Viewpoints. *J. Chem. Educ.* **1985**, *62*, 104.

(19) Einstein, A. On the Motion of Small Particles Suspended in Liquids at Rest Required by the Molecular-Kinetic Theory of Heat. *Ann. Phys.* **1905**, *17*, 549-560.

(20) Stokes, G. G., *Mathematical and Physical Papers*. University Press: Cambridge, 1905.

(21) Galano, A.; Raúl Alvarez-Idaboy, J. Computational Strategies for Predicting Free Radical Scavengers' Protection against Oxidative Stress: Where Are We and What Might Follow? *Int. J. Quantum Chem.* **2019**, *119*, e25665.

(22) Vo, Q. V.; Gon, T. V.; Bay, M. V.; Mechler, A. The Antioxidant Activity of Monosubstituted Indolinonic Hydroxylamines: A Thermodynamic and Kinetic Study. *J. Phys. Chem. B* **2019**, *123*, 10672-10679.

(23) Vo, Q. V.; Mechler, A. In Silico Study of the Radical Scavenging Activities of Natural Indole-3-Carbinols. *J. Chem. Inf. Model.* **2020**, *60*, 316-321.

(24) Okuno, Y. Theoretical Investigation of the Mechanism of the Baeyer-Villiger Reaction in Nonpolar Solvents. *Chem.: Eur. J.* **1997**, *3*, 212-218.

(25) Benson, S., *The Foundations of Chemical Kinetics:* . Malabar, Florida, 1982.

(26) Iuga, C.; Alvarez-Idaboy, J. R.; Vivier-Bunge, A. Ros Initiated Oxidation of Dopamine under Oxidative Stress Conditions in Aqueous and Lipidic Environments. *J. Phys. Chem. B* **2011**, *115*, 12234-12246.

(27) Alvarez-Idaboy, J. R.; Reyes, L.; Mora-Diez, N. The Mechanism of the Baeyer–Villiger Rearrangement: Quantum Chemistry and Tst Study Supported by Experimental Kinetic Data. *Org. Biomol. Chem.* **2007**, *5*, 3682-3689.

(28) Le, T. H.; Tran, T. T.; Huynh, L. K. Identification of Hindered Internal Rotational Mode for Complex Chemical Species: A Data Mining Approach with Multivariate Logistic Regression Model. *Chemom. Intell. Lab. Syst.* **2018**, *172*, 10-16.
